# Effective Removal of Methylene Blue by Surface Alteration of TiO_2_ with Ficus Carica Leaf Extract under Visible Light

**DOI:** 10.3390/nano12162766

**Published:** 2022-08-12

**Authors:** Muhammad Ali Bhatti, Sadaf Jamal Gilani, Aqeel Ahmed Shah, Iftikhar Ahmed Channa, Khalida Faryal Almani, Ali Dad Chandio, Imran Ali Halepoto, Aneela Tahira, May Nasser Bin Jumah, Zafar Hussain Ibupoto

**Affiliations:** 1 Institute of Environmental Sciences, University of Sindh Jamshoro, Jamshoro 76080, Sindh, Pakistan; 2 Department of Basic Health Sciences, Preparatory Year, Princess Nourah Bint Abdulrahman University, Riyadh 11671, Saudi Arabia; 3 Thin Film and Wet Chemistry Lab, Department of Metallurgical Engineering, NED University of Engineering and Technology, Karachi 75270, Sindh, Pakistan; 4 Institute of Physics, University of Sindh, Jamshoro 76080, Sindh, Pakistan; 5 Dr. M.A Kazi Institute of Chemistry, University of Sindh, Jamshoro 76080, Sindh, Pakistan; 6 Biology Department, College of Science, Princess Nourah Bint Abdulrahman University, Riyadh 11671, Saudi Arabia; 7 Environment and Biomaterial Unit, Health Sciences Research Center, Princess Nourah Bint Abdulrahman University, Riyadh 11671, Saudi Arabia; 8 Saudi Society for Applied Science, Princess Nourah Bint Abdulrahman University, Riyadh 11671, Saudi Arabia

**Keywords:** *Ficus carica*, bulk TiO_2_ material, methylene blue

## Abstract

The present study describes the use of a leaf extract from *Ficus carica* as a source of natural antioxidants for the surface alteration of bulk titanium dioxide (TiO_2_) in two steps. First, the hydro-thermal treatment of the bulk TiO_2_ material was carried out and followed by thermal annealing at 300 °C for 3 h in air. The role of the leaf extract of *Ficus carica* on the performance of the bulk TiO_2_ material for the removal of methylene blue (MB) was also studied. Various analytical techniques such as powder X-ray diffraction (XRD), scanning electron microscopy (SEM), and energy dispersive spectroscopy (EDS) were used to explore the crystalline structure, morphology, and composition. The bulk TiO_2_ material after the leaf-extract treatment exhibited mixed anatase and rutile phases, a flower-like morphology, and Ti, O, and C were its main elements. The average crystallite size was also calculated, and the obtained values for the bulk TiO_2_ material, 18.11 nm, and the treated bulk TiO_2_ material with various amounts, 5, 10, and 15 mL, of leaf extract were 16.4, 13.16, and 10.29 nm respectively. Moreover, Fourier-transform infrared spectroscopy validated the typical metal–oxygen bonds and strengthened the XRD results. The bulk TiO_2_ material chemically treated with *Ficus carica* has shown outstanding activity towards the degradation of MB under sunlight. The 15 mL of *Ficus carica* extract significantly enhanced the photocatalytic activity of the bulk TiO_2_ material towards the degradation of MB. The dye degradation efficiency was found to be 98.8%, which was experimentally proven by the Fourier Transform Infrared spectroscopoyy (FTIR) analysis. The obtained performance of the bulk TiO_2_ material with *Ficus carica* revealed excellent surface modifying properties for poorly-performing photocatalysts towards the degradation of synthetic dyes when used in their pristine form. The presented approach suggests that *Ficus carica* could be of great interest for tuning the surface properties of materials, either in the form of nano-size or bulk-phase in a particular application.

## 1. Introduction

The release of wastewater from industries, including pharmaceuticals, textile, chemical, sugar, beverages, paper, and so forth, is accompanied by several organic and inorganic pollutants, which severely deteriorate the resources of natural water [1,2]. The published works confirm that many of the synthetic dyes, such as methylene blue, acid black 234, mordant black 1, acid black 210, etc., are life-threatening to us [3]. It has been found that the degradation of synthetic dyes is a big challenge to less toxic compounds [4]. Hence, it is an immediate task for scientists and environmentalists to develop either new or modified methods for the efficient degradation of synthetic dyes [5]. The existing commercial methods, including biological and physiochemical for wastewater treatment, have a performance for the removal of organic pollutants. These methods have certain demerits, such as high cost, the consumption of a lot of energy, poor performance at low concentrations of dye, and the poor decolorization of dyes [6,7]. The use of advanced oxidation processes mainly depends on the generation of active OH radicals, which efficiently degrade organic pollutants [8,9]. Photocatalysis is among the advanced oxidation processes; it is highly studied due to its potentiality for the mineralization of organic pollutants into carbon dioxide and water [10,11]. For this purpose, various photocatalysts such as titanium dioxide (TiO_2_), ZnO, ZnS, Fe_2_O_3_, and graphene composite have been investigated. The use of TiO_2_, compared to other materials used as photocatalysts, is studied intensively because of the low toxicity, cost, and strong oxidizing capability [12]. In contrast to this, the wide band gap of pristine TiO_2_ has limited its performance up to the UV light, which is only 5% of the solar spectrum and does not capitalize the visible light, approximately 45% of the solar light. Furthermore, the recombination of the rate of electron and hole pairs is high in the bulk TiO_2_ material, which impairs the degradation activity. Therefore, it is very important to fabricate the visible light photosensitive materials in order to capitalize the full solar spectrum and enhance the photocatalytic efficiency [13,14]. Different methodologies for the surface tuning of TiO_2_ towards visible light functionality, such as metal dopants [15], nonmetal dopants [14], hybrid composites [16,17], dye sensitizing methods [18], and dyes [19], have been used. However, there has been little attention on the use of natural antioxidants for the surface modification of bulkTiO_2_ nanoparticles, thus increasing the degradation performance in the visible region. *Ficus carica* (FC) grows in tropical and subtropical climates around the world. It is a member of the Moraceae family and is a rich source of many bioactive substances (phenolic compounds, vitamins, minerals, carbohydrates, dietary fibers, sugars, and organic acids) that are low in fat, cholesterol, and have high amino acid content. Leucoderma and ringworms were traditionally treated using the root of *Ficus carica* plant. The fruit is sweet and has antipyretic properties of aphrodisiac, and has proven useful in paralysis and inflammations. The FC leaves are used to cure jaundice [20]. The chemical components of FC include fatty acids, stigmasterol, fucosterol, campesterol, and β-sitosterol [21], as well as psoralen, umbelliferone, and bergapten [22,23], calotrophenyl, and lupeol acetate [24,25,26], and 6-O-acyl-d glucosyl-sitosterol as an antiproliferative agent The anticancer compounds 9,9-cycloarlane triterpenoid and 6-(2-methoxy-Z-vinyl)-7-methyl-pyranocoumarin glucosyl-sitosterol [26,27]. Among the natural oxidants, fig (*Ficus carica*) leaf extract is rich with polyphenols, flavonoids, and a high density of antioxidants [28]. The leaf extract of *Ficus carica* is neither utilized for the surface alteration of bulk TiO_2_ material nor is it used for the degra dation purposes of MB under natural sunlight to date. As a result, we looked into how *Ficus carica* (FC) leaf extract affected the ability of bulk TiO_2_ to degrade MB when exposed to sunlight naturally. The advantages of the present study are the simplicity, low cost, and the surface modification of the bulk TiO_2_ material is performed by an abundant natural source of antioxidants from *Ficus carica*. Moreover, the present approach is very simple, and it prevents the toxic effects of nanostructured materials just by using the bulk TiO_2_ material with excellent degradation efficiency towards MB in an aqueous solution.

In this study, we have used bulk TiO_2_ material and chemically treated it with a leaf extract of *Ficus carica* (FC) via a hydrothermal process followed by thermal annealing at 300 °C in air. The material characterization was carried out through SEM, EDS, XRD, and UV–visible spectroscopy techniques. The photocatalytic activity of the bulk TiO_2_ chemically treated with *Ficus carica* (FC) was studied with the degradation of MB under natural sunlight. The obtained degradation efficiency was around 98.8% at 240 min.

## 2. Materials and Methods

### 2.1. Chemical Reagents

The FC leaves were obtained from the garden of the Institute of Chemistry, University of Sindh Jamshoro. The model dye methylene blue (C_16_H_18_ClN_3_S, Mm = 319.85 g/mL), Titanium dioxide (Mm = 79.866) as bulk, ammonium hydroxide (Mm = 35.04, 25% NH₄OH) were purchased from Merck). Excellent analytical grade chemicals were utilized in a received condition. For material synthesis and the preparation of the stock solution of methylene blue, deionized (DI) water was used thoroughly.

### 2.2. Preparation of Leaf Extract

At first, the leaves obtained from the FC plants were thoroughly washed with tap water followed by distilled water to ensure the removal of the dirt. About 15 g of leaves were ground by a mortar and pestle, resulting in a paste. Next, the paste was then placed in 50 mL of DI and annealed for 25 min at 65 °C. The mass was then filtered by using filter paper. Finally, the resultant leaf extract was applied as an agent for surface modification for the TiO_2_.

### 2.3. The Surface Alteration of Bulk TiO_2_ Material by Ficus Carica (FC) Leaf Extract

At first, four beakers were prepared to bear a solution composed of (2.22 g) of titania powder and 5 mL of 25% ammonium hydroxide. Then, 100 mL of DI water and four different compositions of FC plant leaf extract (5 mL, 10 mL, and 15 mL) were added to three beakers. The designation “pristine titanium dioxide” was given to the fourth beaker, which was left in its prepared state (TiO_2_). The aluminum foil was used to completely enclose the beakers, which was performed after 5 h of preheating the oven to 95 °C. After annealing, the bulk TiO_2_ appeared as a white powder on the filter paper and was ready for further characterization.

### 2.4. Characterization

At first, the morphological investigation of modified TiO_2_ was performed by SEM at an accelerating voltage of 10 kV. In contrast, the elemental composition of treated TiO_2_ was performed by Energy dispersive X-ray spectroscopy (EDS) (model Jeol JSM-6380 A, Tokyo, Japan). At 45 kV, CuK radiation (=1.54050), and 45 mA, a powder X-ray diffractometer (XRD) (PANlytical, Netherland) was used to study the purity and phase analysis of extract-treated bulk TiO_2_ material. The T–O bond was examined using FTIR equipment (Spectrum two PerkinElmer, Waltham, MA, USA), and dye degradation using the changed bulk TiO_2_ material and *Ficus carica* (FC) leaf extract was demonstrated [29].

### 2.5. Photo Catalytic Measurements

Methylene Blue was selected as a model pollutant for examining its photocatalytic degradation by using the chemically treated bulk TiO_2_ material. The photocatalytic activity was performed under natural sunlight irradiation. At first, 50 mL of 5 × 10^−5^ M solution of Methylene Blue dye with a dye concentration of 1 mg/50 mL and 2 mg/50 mL of pristine TiO_2_ and surface treated TiO_2_ in conjunction with FC leaf extract 5, 10, and 15 mL were introduced in four distinct glass beakers. The mixture was then sonicated for five minutes in the dark to guarantee uniformity. Next, the solution was exposed to sunlight at predetermined intervals, such as 30–360 min (for 6 h). The UV–vis spectrophotometer (PE Lamada 356) was used in the optical experiment to look at the dye absorption spectra during the photocatalytic degradation process. According to the analysis, the reaction mixture of absorbance reduced as the exposure duration increased, which indicates that the concentration of MB dye decreased as well. The photocatalytic degradation was carried out, and the calculations were made under the following formula:

Dye elimination % (*C*_0_ − *C*_t_)/*C*_0_ × 100 (1)

where, *C*_0_ stands for the dye’s initial concentration and *C*_t_ for the dye’s concentration after following the application of sunlight, respectively.

## 3. Results and Discussion

### 3.1. Analysis of Structure, Phase and Composition of the Surface Modified Bulk TiO_2_ Material after Treated with FC Leaf Extract

#### 3.1.1. Morphological Investigation

**Figure 1:**
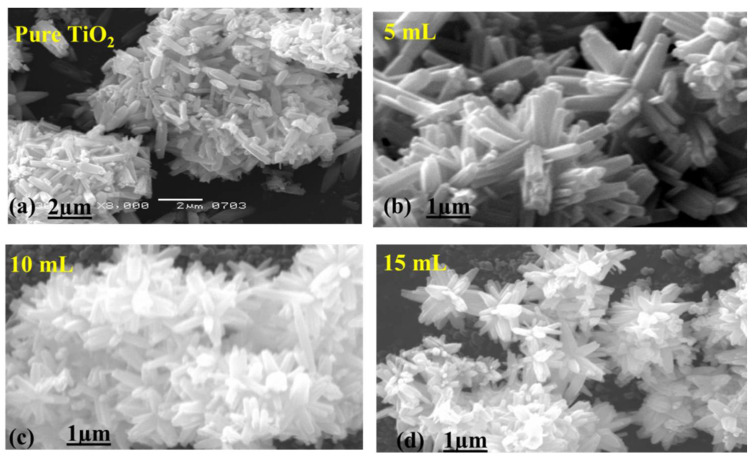
SEM images of bulk TiO_2_ that has been hydrothermally treated (**a**) and treated with 5, 10, and 15 mL of FC leaf extracts (**b**–**d**).

**Figure 2:**
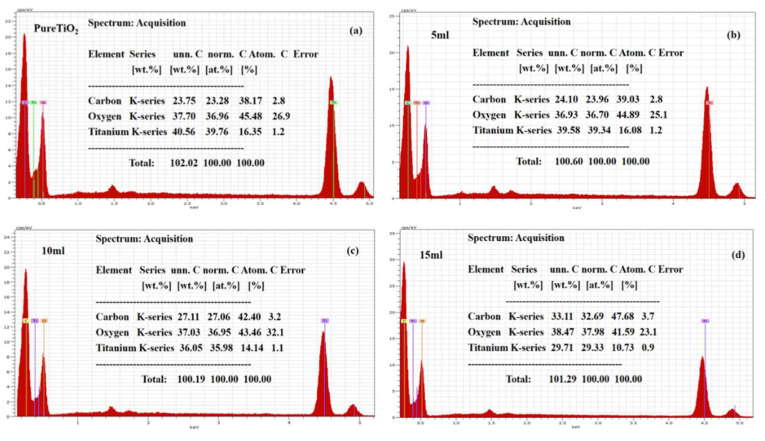
EDX analysis of bulk TiO_2_ that has been hydrothermally treated (**a**) and treated with 5, 10, and 15 mL of FC leaf extracts (**b**–**d**).

Using the SEM technique, the morphology of the surface-modified and pure TiO_2_ was studied. It was observed that the pristine bulk TiO_2_ was bearing a rod-like structure having a dimension in microns, as given in Figure 1a. Whereas the samples treated with 5 mL of leaf extract seemed to bear little variation in shape with a flower-like appearance [30], next, the samples with 10 and 15 mL of leaf extract followed a similar trend of bearing a flower shape with a slightly thinner surface, as presented in Figure 1c,d. A little difference in morphology appeared at a certain value after the addition of leaf extract to TiO_2_ material, which may be corresponding to the formation of electrical charges. The chemical composition for pristine TiO_2_ material and bulk leaf extract treated TiO_2_ material is enclosed in Figure 2a–d. The samples throughout revealed that the composition was mostly dominated by Ti and O, as depicted in Figure 2a–d.

The use of the FC extract has shown a significant effect on the morphology, as can be seen from SEM images, and it can play a vital role in dye degradation kinetics. We have observed that the surface of bulk TiO_2_ became thinner with the use of higher amounts of FC extract, and it can further expose more surface area with catalytic sites for light interaction. Consequently, a high-density generation of electrons and holes further adds high-value oxidizing radicals such as hydroxyl and superoxide. Then, these oxidizing radicals participate effectively in reducing the MB at high degradation effectively.

#### 3.1.2. XRD Investigation

**Figure 3:**
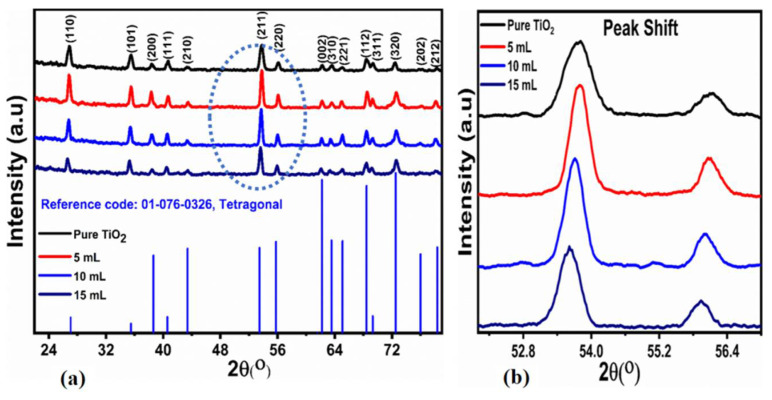
XRD patterns bulk TiO_2_ that has been hydrothermally treated (**a**) and treated with 5, 10, and 15 mL of FC leaf extracts (**a**) and peak shift analysis at (211, 220) with increasing amount of *Ficus carica* (FC) (**b**).

The diffraction peaks of the pure and leaf-extract-treated samples were examined by the XRD technique. The resultant peaks were in good agreement with the reference ICSD card no.01-076-0326, bearing a tetragonal crystalline structure with a mixed phase of anatase and rutile. As illustrated in Figure 3a, the appearance of various diffraction patterns at two specific theta angles are observed at 27.05° (110), at 35.48° (101), at 38.63° (200), at 40.57° (111), at 43.41° (210), at 53.46° (211), at 55.78° (220), at 61.58° (002), at 62.21° (310), at 65.09° (221), at 68.43° (112), at 69.31° (311), at 72.52° (320), at 76.00° (202) and at 78.33° (212). These reflection peaks indicate the excellent crystalline aspects of the treated samples with FC extract.

The resultant diffraction peaks at 2θ were recorded at 27.04° (110) and 43.41° (210) corresponded to the rutile phase of bulk titania. The rest of the peaks corresponded to the anatase phase belonging to bulk TiO_2_. These observations suggest that the material is mainly characterized by mixed phases of anatase and rutile. The presence of mixed phases with rutile and anatase bulk TiO_2_ material corresponding to leaf extract might be assigned to the variation in the oxygen concentration. It was further noticed that oxygen was significantly impressed by the synthesis technique and the resultant crystalline phase [31]. At the plane (211), the peaks are highly intense, showing the predominantly-favored crystal formation leading to a countable shift of reflection peak could be observed in Figure 3b. The reason for the shift might be assigned to the smaller ionic radii of titanium dioxide. According to Sagneetha et al., another possible cause of the minor shift to the lower angle is a change in crystal size and morphological disorder [32].

The average crystalline size for the pristine and leaf-extract-treated samples was estimated by using Debye–Scherrer’s Equation (2) at (211) an intense diffraction peak. The crystalline diameters of all of the samples, whether untreated or treated, were found to range between 10.29 and 18.11 nm, as indicated in Table 1. The structural details of each sample are also listed in Table 1.

(2) $$\mathrm{Dp}=\frac{0.94\lambda }{\beta_{1/2}\cdot \cos\theta }$$

Here, D_p_ denotes the mean crystalline domain, which can be greater or smaller than a particle size of less than 100 nm, λ indicates the X-ray wavelength of CuKα radiation (1.54 Å), β_1/2_ represents full width and half maximum. According to Table 1, the bulk TiO_2_ material treated with various quantities of FC leaf extract had an average crystallite size of 18.11 nm, 16.14 nm, 13.16 nm, and 10.29 nm.

**Figure 4:**
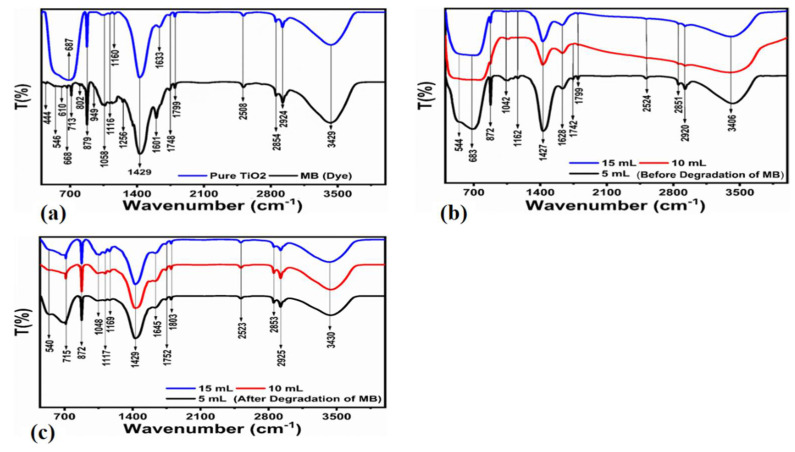
FTIR spectrum of bulk TiO_2_ that has been hydrothermally treated and MB (**a**), FTIR spectrum of TiO_2_ treated with 5, 10, and 15 mL of FC leaf extract before degradation of MB (**b**) and FTIR spectrum of TiO_2_ treated with 5, 10, and 15 mL of FC leaf extracts before degradation of MB (**c**).

Table 1 clearly shows that the treated leaf extract TiO_2_ material has a smaller size than untreated TiO_2_. Furthermore, the catalyst effect on photocatalytic activity is assigned to the crystalline size and surface area. Therefore, if the crystalline size is smaller, the surface area will be larger, and photodegradation activity will be higher [33]. The FTIR technique was used to identify functional groups and to investigate the residual organic compounds in the extract-treated samples, as given in Figure 4. The FTIR spectrums for the untreated TiO_2_ as well as methylene blue are depicted in Figure 4a. Corresponding peaks in the range of 3429 cm^−1^ and 2508 cm^−1^ represent the vibrational modes of the –OH group. Matching frequencies for the bulk untreated TiO_2_ were detected at 687 cm^−1^ to confirm the typical metal–oxygen vibration modes, as in our investigation, and peaks at 1633 cm^−1^ to demonstrate the unique Ti–O–Ti bonding characteristics of the bulk TiO_2_ crystal [34]. As shown in Figure 4b, FTIR analysis of TiO_2_ treated with different leaf extract concentrations was also performed. The majority of the metal–oxygen peaks were discovered to have persisted in all samples with only a minor shift in frequency, confirming the high purity of the samples and were in good agreement with the XRD findings. Furthermore, the breakdown of dye by the leaf-extract-altered material was affirmed by the FTIR results, as given in Figure 4c. It is explicit that most of the vibration modes for the pollutant vanished during the degradation process. This proves the excellent efficiency of the treated samples. The variations at 715 cm^−1^ and 2853–2925 m^−1^ could be connected to the C–H bending vibrations that emerged from the possible negligible number of MB molecules after the degradation process.

Figure 4b,c include the IR spectra before and after the degradation of MB, respectively. The spectra are closely the same, and we aim to show through IR study the successful degradation of MB near the surface of the presented photocatalysts. However, the confirmation of the modification to bulk TiO_2_ should be verified before, which is difficult to show in the presented work. Therefore, new studies are required to investigate this part of the presented photocatalysts in the future.

### 3.2. Photocatalytic Efficiency

**Figure 5:**
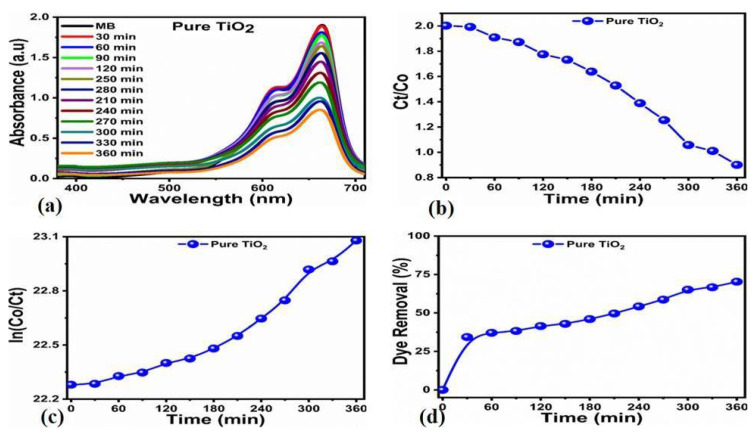
Changing in UV–vis spectra of pure TiO_2_ (**a**), plot of Ct. Co verses irradiation of photodegradation of MB at various times (**b**), pseudo-first order plot for photodegradation of MB (**c**) and shows the calibration curve of dye removal (%) under natural light illumination of only hydrothermally treated bulk TiO_2_ material (**d**).

From the reaction mixture, an aliquot of 3 mL was taken at certain intervals, followed by a measurement of the absorbance of MB at 664 nm. A decrease in the intensity of the absorption was observed continuously, corresponding to the degradation of MB. The UV–vis spectra for the untreated TiO_2_ with a concentration of 2 mg in MB dye (5 × 10^−5^ M) under sunlight for various time periods from 30 min to 360 min at an equal interval of 30 min is displayed in Figure 5a.

The results showed that for the samples without the leaf-extract treatment, the dye degradation occurred on the surface. The concentration (Ct./Co) for MB is displayed in Figure 5b. The untreated TiO_2_ followed reaction kinetics in good agreement with the pseudo-first-order, as shown in Figure 5c. The dye removal percentage (%) results are given in Figure 5d. A poor degradation efficiency trend was noticed for the untreated TiO_2_. The maximum degradation efficiency of 70.31% was recorded for the degradation time of 360 min (Figure 5d).

**Figure 6:**
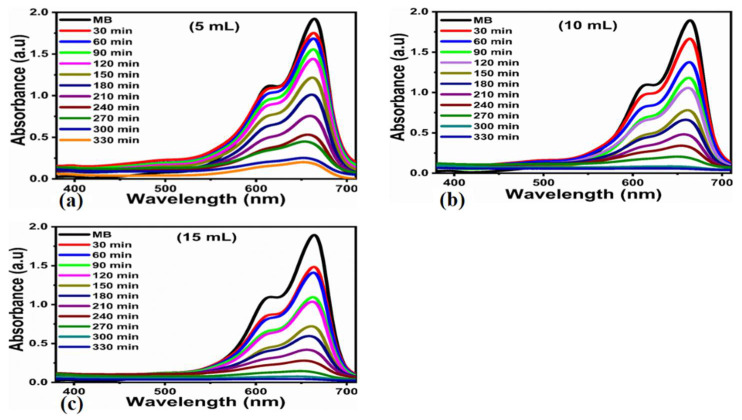
(**a**–**c**) change in Uv–Vis spectrum for photodegradtion of MB under the natural light illumination over bulk TiO_2_ that has been hydrothermally treated with 5, 10, and 15 mL of FC leaf extracts at 30–330 min.

The poor efficiency could be attributed to fast electron-hole pairs recombination and the lack of catalytic sites for the untreated TiO_2_ material. The UV–vis spectrum of 1 mg of the TiO_2_ material with various doping concentrations of FC leaf extract of 5, 10, and 15 mg were examined in MB dye (5 × 10^−5^ M) under sunlight irradiation for times ranging from 30 min to 33 min at an equal interval of 30 min up to the maximum time of 330 min, as depicted in Figure 6a–c.

**Figure 7:**
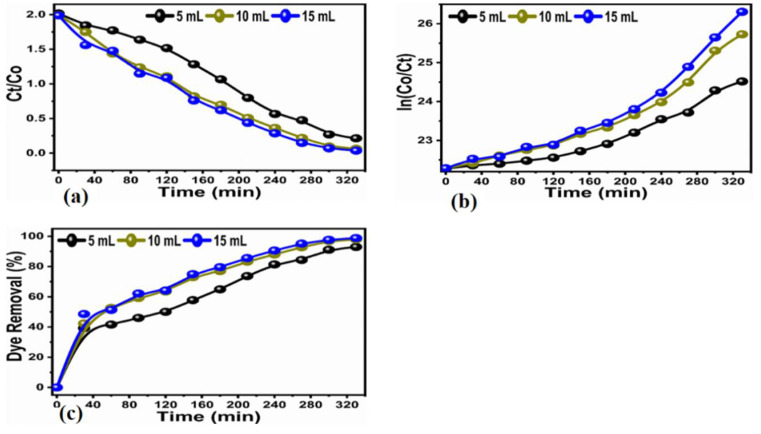
Plot of Ct. Co verses irradiation of photodegradation of MB at various times (**a**), pseudo-first order plot for photodegradation of MB (**b**) and the calibration curve of dye removal (%) undernatural light illumination of bulk TiO_2_ material that has been hydrothermally treated with 5, 10, and 15 mL of FC leaf extracts at 30–330 min (**c**).

The kinetics of photodegradation for FC leaf-extract-treated TiO_2_ seemed to be following pseudo-first order reaction kinetics, as shown in Figure 7a. This is because the rate of reaction is related to the concentration of the dye, and the reaction degradation was performed in an aqueous solution. The entry of water tells us that the rate of the reaction is not dependent on it; this is the reason we believe that reaction kinetics are mainly governed by pseudo-first-order reaction kinetics.

With respect to time, a rising tendency of deterioration efficiency was seen. For 5 mL of the FC leaf-extract-treated TiO_2_, an excellent maximum efficiency of 92.95% was achieved. The concentration was increased to 10 mL for the next sample, an increase in the efficiency trend was observed, and enhanced efficiency of up to 97.89% was recorded. The next sample with an increased FC leaf extract of up to 15 mL in TiO_2_ resulted in a further increased efficiency for the removal of MB of up to 98.87%, as depicted in Figure 7c.

**Figure 8:**
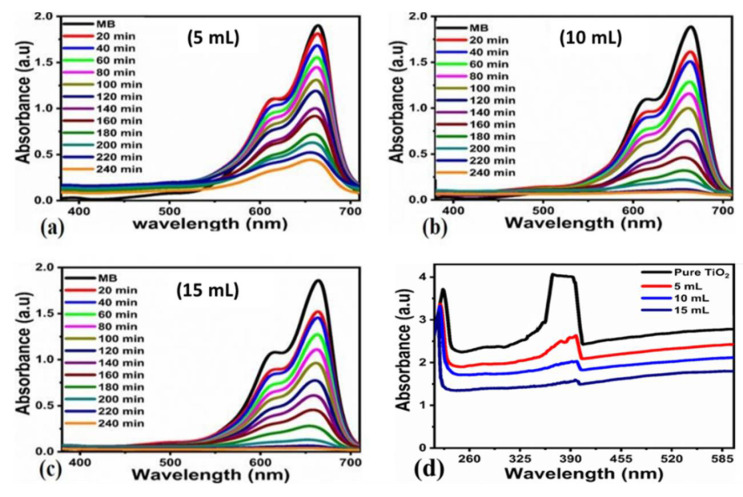
(**a**–**c**) change in Uv–Vis spectrum for photodegradtion of MB under the natural light illumination over bulk TiO_2_ that has been hydrothermally treated with 5, 10, and 15 mL of FC leaf extracts at 20–240 min. (**d**) A comparison of absorbance of pristine Titania with doped ones.

It is very evident from the results that the pollutant MB was almost 100% removed by using a minute amount (1 mg) of bulk titania treated with FC leaf extract with time variations. Comparatively, varying amounts (2 mg) of bulk titania treated with varying amounts of FC leaf extract, namely (5, 10, and 15 mL), were measured using the availability of sunlight treatment for time variations of 20 to 240 min at equal intervals of 20 min, as shown in Figure 8a–c. The absorbance spectra for pristine and modified TiO_2_ is also presented in Figure 8d, which corresponds a variation in the absorbance with respect to the increase in dopant dose.

**Figure 9:**
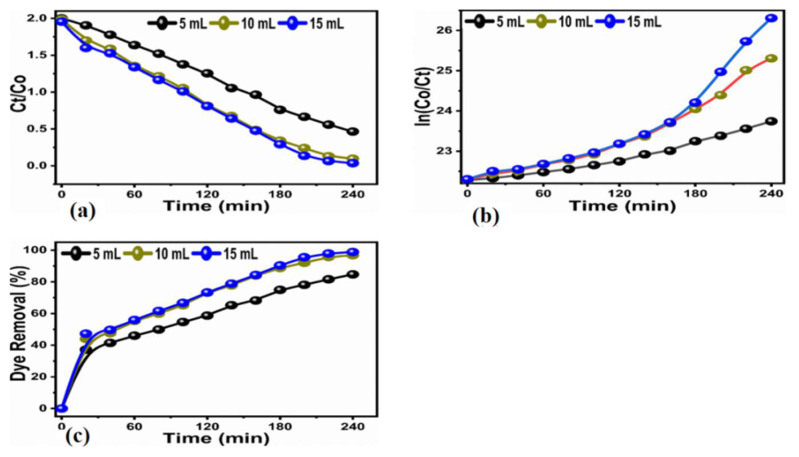
(**a**) Plot of Ct. Co verses irradiation of photodegradation of MB at various times (**b**), pseudo-first order plot for photodegradtion of MB (**c**) and the calibration curve of dye removal (%) under natural light illumination of bulk TiO_2_ material that has been hydrothermally treated with 5, 10, and 15 mL of FC leaf extracts at 20–240 min.

Pseudo-first and second-order schemes, as shown in Figure 9a,b, were explored to better understand the kinetics of the degradation of the bulk TiO_2_ that had been treated using the FC leaf extract. The efficiency of pollutant removal was examined with respect to varying time intervals for up to 240 min. The minimum efficiency of 37.20% was recorded with an increasing trend up to the maximum efficiency of 84.70% was recorded for the 5 mL of leaf-extract altered TiO_2_, 96.80% was achieved for 10 mL and 98.82% for 15 mL, as depicted in Figure 9c. Therefore, it is clear from the obtained results that adding more FC leaf extract into the synthesized material will significantly increase the photocatalytic activity of bulk TiO_2_ for the removal of the organic dye contaminant MB. The density of the antioxidants was significantly altered by the addition of a concentration of the FC leaf extract for the degradation of Methylene blue. Increasing the FC leaf extract affects the density of antioxidants highly [35]; therefore, it has highly impressed the surface properties of TiO_2_ with respect to morphology, crystal size, as well as the number of catalytic sites on the material surface that favored a superior efficiency of degradation. The result of the 15 mL leaf-extract-treated bulk TiO_2_ provided superior degradation behavior for the successive removal of pollutants. Different FC extract amounts have shown a certain effect on the change in morphology, and with a larger amount of FC extract, the surface of TiO_2_ became much sharper, suggesting that its larger surface area favored photocatalytic properties.

Finally, the kinetic study for the concentration of the dye was observed by a Langmuir–Hinshelwood (L-H) model [36,37], as shown in (Figure 7a and Figure 9a).

The efficiency of dye degradation efficiency was calculated by:

*C*/*C*_0_ = *A*/*A*_0_ (3)

where *A*_0_ as well as *A* represents absorbance recorded before and after the reaction for the degradation of methylene blue at 664 nm. The following model may describe the statistics more significantly.

Ln(*C*/*C*_0_) = −K_app_t (4)

**Figure 10:**
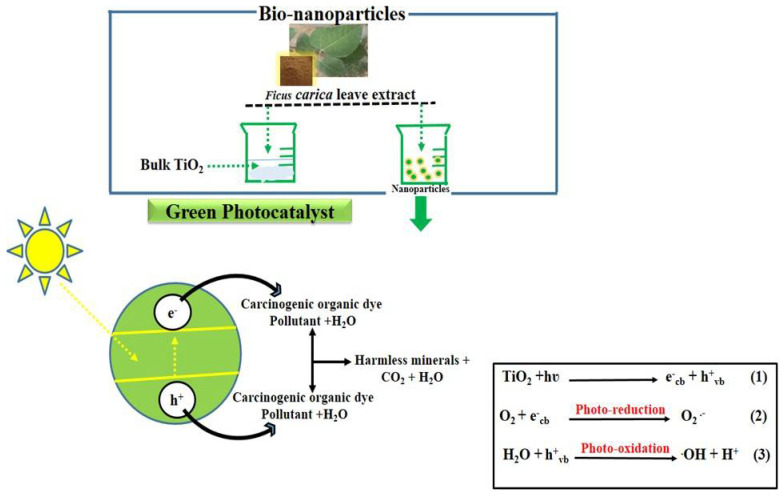
Projected mechanism of photocatalytic degradation of dyes using bulk TiO_2_ material treated with various *Ficus carica* leaf extract of 5, 10, and 15 mL.

In this case, K_app_ stands for apparent velocity constant for the dye degradation process. Where as *C* and *C*_0_ shows the initial dye concentration and dye concentration after certain intervals of time. As enclosed in (Figure 8b and Figure 10b). The correspond rate constants for the two different dye concentrations and various amounts of FC extract, such as 5 mL (K = 6.8410^−3^ min^−1^, K = 6.13 × 10^−3^ min^−1^), 10 mL (K = 9.99 × 10^−3^ min^−1^, K = 1.23 × 10^−2^ min^−1^), and 20 mL (K = 9.99 × 10^−3^ min^−1^, K = 1.23 × 10^−2^ min^−1^).

As a typical semiconductor metal oxide photocatalyst, TiO_2_ produces (e^−^ and h^+^) when illuminated by light energy greater/equal to the band gap width. Some of these pairs are migratory, and the redox (reduction–oxidation) reaction involves interactions with absorbed species on the surface of the nanocatalyst. The MB degradation is mediated by the radicals produced by the generated electron and hole pairs after the interaction of solar light with the surface of TiO_2_. Radicals such as hydroxyl and superoxide are generated by the interaction of electron and hole pairs with water and oxygen from an aqueous solution of the dye. Additionally, as shown in the equations, the absorbed organic molecules are removed when h^+^ vb interacts with H_2_O and O_2_ from the aqueous solution of MB to form hydroxyl (OH) and (e^−^ cb) continuously consumes oxygen to form a superoxide radical (O^2−^) anion as given (Figure 10). In the degradation of MB under sunlight, the hydroxyl and superoxide radicals are the main radicals which favor the conversion of MB into mineralized products, including carbon dioxide.

Figure 10 depicts a proposed mechanism for the breakdown of dye methylene blue when different concentrations of FC leaf extract are mixed with the bulk TiO_2_ during exposure to sunlight. As is customary, hazardous organic pollutants are broken down into innocuous byproducts such as (CO_2_, H_2_O) or other organic ions by diverse catalytic pathways in nanostructured materials [38]. The acquired findings were also compared, and the observed performance of the proposed photocatalyst is included in Table 2. The performance evaluation confirms that the proposed photocatalyst could be considered for wastewater treatment as it is associated with superior or equal performance to recently reported photocatalysts [39,40,41,42,43,44,45,46,47,48,49,50,51,52,53,54,55,56], and the material fabrication is low cost and simple, which enables large-scale production.

## 4. Conclusions

In summary, we have used the leaf extract of *Ficus carica* for the surface modification of bulk TiO_2_ material by a two-step methodology. First, the bulk TiO_2_ was treated with different amounts of leaf extract of *Ficus carica* using the hydrothermal method. Second, the hydrothermal leaf extract treated with bulk TiO_2_ material was combusted at 300 °C for 3 h in order to remove the residual products from the sample.

There was little effect on the morphology of the material; however, with the increasing amount of leaf extract of *Ficus carica*, the top surface of the material became sharp. The crystalline aspects were found in the mixed phase of anatase and rutile for TiO_2_. The average crystallite size of TiO_2_ was noticed to be smaller than bulk TiO_2_ without treatment_._ The *Ficus carica* treated bulk TiO_2_ material was found to be highly active towards the degradation of MB under natural sunlight, and a degradation efficiency of 98.8% was observed for 240 min. The enhanced degradation performance of the leaf-extract treated bulk TiO_2_ material is attributed to the minimum charge recombination rate, tuned surface features, and decrease in crystallite size. These findings confirm that the *Ficus carica* leaf extract containing a natural source of antioxidants can be of high privilege as a surface-modifying agent for a wide range of photosensitive materials.

## Figures and Tables

**Table 1:** Structural properties of bulk TiO_2_ i-e hydrothermally treated and altered with 5, 10, and 15 mL of FC leaf extract.

Sample	2θ (°) (211)	FWHM	Height	Crystalline Size (nm)	Constant (K) 1 mg	Constant (K) 2 mg
Pure TiO_2_	53.81	0.55333	36.55	18.1	-	2.27 × 10^−3^ min^−1^
5 mL	53.78	0.37074	51.55	16.1	6.84 × 10^−3^ min^−1^	6.13 × 10^−3^ min^−1^
10 mL	53.71	0.38377	51.33	13.1	9.99 × 10^−3^ min^−1^	1.23 × 10^−2^ min^−1^
15 mL	53.62	0.41464	38.77	10.2	1.14 × 10^−2^ min^−1^	1.55 × 10^−2^ min^−1^

**Table 2:** The comparative analysis of presented results with published works.

Catalysts		Weight	Light Source	Dye Concentrati on	Time (min)	Dye Removal (%)	Method	Ref.
TiO_2_@Bi_2_O_3_		35 mg	Sun light	2 mg of catalyst (50 mL of dye solution)	250	94%	Green synthesis	[39]
TiO_2_ NPs		100 mg	UV irradiation	100 mL MB	120	92%	Chemical and green synthesis methods	[40]
TiOSO_4_		0.1 g	UV irradiation	20 ppm MO	150	94%	Sol-gel method	[41]
MnTiO_3_		5 mg	Sun light	MB 1 × 10^−5^ M	250	75%	Sol-gel	[42]
Fe_2_TiO_5_		50 mg	Sunlight	MB 10 mg L^−1^	250	97%	Sol-gel	[43]
porous TiO_2_ ceramic s		eighty pieces (20 mg each piece)	UV light	RhB 10 mg L^−1^	300 min	99.3%	camphene-based freeze-casting process	[44]
macro/mesop orous anatase TiO_2_ ceramic		eighty pieces (20 mg each piece)	UV light	RhB 10 mg mL^−1^	180	99.4%	a camphene-based freeze-casting process	[45]
Fe–N- codoped TiO_2_/fly ashecnospheres		0.2 g	Sun light	RhB mg L^−1^	4 h	89%	Sol-gel method	[46]
hollow TiO_2_/fly ashecnospheres		1.5 g	Sun light	MB 30 mg L^−1^	9 h	0.0922 (min^−1^)	Sol-gel	[47]
Ag^+^-doped TiO_2_/ploystyrene		0.05 g	Sun light	MB 5 mg L^−1^	5 h	83%	Impregnation and strewing based on a simple solvent-cast method	[48]
TiO_2_/polyuret hane foam		forty pieces	Sun light	MB + Cr^VI^ 1 0 mg L^−1^	150	90%	a low-temperature ultrasonic and deposition approach	[49]
TiO_2_/low density polyethylene		1 g	Sun light	MB 0.16 mmol L^−1^	225	30%		[50]
C,N- TiO_2_/polytetrafluoroethylene		1 gL^−2^	Visible light	MO 20 mg L^−1^	4 h	96.9%	y a simple high-energy ball-milling process	[51]
B–N– TiO_2_/expanded perlite		(6 mg/g, 24 wt% TiO_2_	Solar light	RhB	5 h	99.1%	Sol-gel	[52]
W doped ZnO		20 mg	UV light	MB	3 h	96.9%	Low temperature aqueous chemical growth method	[53]
TiO_2_- graphene		0.2 g	UV light	RhB 0.02 mM	2–4 h	95%	Sol-gel	[54]
TiO_2_/polypro pylene		15– 20 mg	UV light	MO 15 mg L^−1^	4 h	65%	Low temperature Hydrothermal method	[55]
TiO_2_/polypro pylene fabric		a piece (d = 47 mm)	UV light	MO 15 mg L^−1^	240	100%	Hydrothermal Process	[56]
BC-TiO_2_		---------	UV light	MB 10 mg/L	180	90%	Low temperature Pyrolysis	[57]
TiO_2_/AC		10 g	Sun light	25 mg dm^−^^3^	90	98.1%	in situ immobilization process	[58]
TiO_2_/CRFs		10 mg	Solar light	10 mg L^−1^	80	98.1%	Impregnation and Calcination method	[59]
TiO_2_	Ficus	2 mg	Solar Light	5 × 10^−5^ M (MB)	330	98.82%	Simple Hydrothermal Process	Present Work
carica leave extract								

## Data Availability

Not applicable.
